# Development and Validation of Nomogram-Based Prognosis Tools for Patients with Extremity Osteosarcoma: A SEER Population Study

**DOI:** 10.1155/2022/9053663

**Published:** 2022-05-12

**Authors:** Yingtao Huang, Chenchen Wang, Dadong Tang, Bing Chen, Zhongchao Jiang

**Affiliations:** ^1^Chengdu University of Traditional Chinese Medicine, Chengdu, 610075 Sichuan Province, China; ^2^Hunan University of Traditional Chinese Medicine, Changsha, 410208 Hunan Province, China

## Abstract

**Objective:**

Osteosarcoma, usually occurring in the extremities, is the most common malignant bone tumour. The purpose of this study is to develop and validate nomogram-based prognosis tools for survival (OS) and cancer special survival (CSS) of patients with osteosarcoma of the extremities via the application of survival analysis.

**Materials and Methods:**

A total of 1427 patients diagnosed with osteosarcoma of the extremities during 2004–2015 were selected from the National Cancer Institute's (NCI) Surveillance, Epidemiology, and End Results- (SEER-) Medicare database. The samples were randomly assigned to either the training set (*n* = 856) or the validation cohort (*n* = 571). Kaplan–Meier (K–M) survival analysis was conducted to calculate patients' 1-, 3-, and 5-year OS and CSS rates. Cox proportional hazard ratio (HR) regression models were employed to identify and examine the factors that have a significant impact on OS and CSS with data from the training cohort.

**Results:**

The results of univariate and multivariate analyses performed in the training cohort indicated that older age, increased tumour size, higher grade, distant tumour extension, amputation, or no surgery (all HR > 1, *P* < 0.05) were risk predictors of poor OS and CSS. Subsequently, the independent prognosis signatures were utilised to construct nomograms. The concordance index (C-index), calibration plot, and decision curve analysis (DCA) were simultaneously used to validate the nomograms. The internally validated C-index values of the OS and CSS prediction models for the training set were 0.752 (95% confidence interval [CI]: 0.738–0.765) and 0.754 [95% CI: 0.740–0.768], respectively. Then, the models were validated in the validation cohort population, which also demonstrated the models had good reliability for prognostication.

**Conclusions:**

The SEER cohort of patients with osteosarcoma of the extremities can be employed to produce effective tools that can assist in prognosis modelling.

## 1. Introduction

Osteosarcoma, or osteogenic sarcoma, is the most frequent aggressive primary malignant tumour of bone in children and adolescents, as well as the third most frequent in adults [1]. In addition to that, there is a second incidence peak among senior adults aged 70–90 years [2]. Generally, around 80% of osteosarcomas occur in the extremities [3], and femur, tibia, and humerus account for approximately 85% of extremity tumours [4].

Osteosarcoma was once recognised as a death sentence. Before 1970, treatments for tumours were mainly composed of surgical resection [5]. At that time, the survival rate for patients with osteosarcoma in the U.S. was only about 20% [6]. In the early 1980s, clinical trials examining the efficacy of chemotherapy began to emerge. Those medical studies, which involved chemotherapy given both before and after definitive tumour resection, led to a rapid increase in the 5-year survival probability to around 70% [2]. However, accurate predictive models allowing individualised estimation of survival probabilities for patients with osteosarcoma of the extremities are now lacking.

The Surveillance, Epidemiology, and Outcomes (SEER) database of the National Cancer Institute (NCI) has collected detailed cancer patients' demographic and clinical information covering the date of diagnosis, tumour stage, treatment, and mortality [7, 8]. This study was conducted for the purpose of determining the prognostic factors associated with primary osteosarcoma of the extremities. As such, patient demographics, tumour characteristics at the time of diagnosis, and treatment variables recorded within a SEER dataset were introduced and evaluated against the outcomes of overall and cancer-specific survival. Then, the significant predictors were used to construct nomogram-based prognostic prediction models. Furthermore, the reliability of the predictive models was validated both internally and externally.

## 2. Materials and Methods

### 2.1. Research Aim

The study design comprised a retrospective cohort study conducted from a predictive perspective using a time-to-event (survival analysis) methodology. The patients diagnosed with osteosarcoma of the extremities during 2004–2015 were identified from the SEER-18 Registries database. This investigation not only examined influential factors for OS and CSS but also aimed at establishing nomogram-based prognosis tools that can provide individualised survival projections based on demographic and cancerous tumour-associated and treatment-related factors.

### 2.2. Patient Data Collection and Selection

SEER data are widely considered robust and valid [9, 10]. According to the working members of the SEER program, there is about 98% completeness in the reported data within 22 months after diagnosis, and annual audit reports of the data are issued to supervise and ensure accuracy [10, 11]. The SEER program's 18 (SEER-18) Registries Database from 2000 that covers 27.8% of the total population in the US [12] provides a considerable amount of credible clinical data of patients with long and short bones of the upper and lower extremities.

During the years 2004–2015, there were 2229 cases of primary extremity osteosarcoma in the SEER-18 Registries Database. The dataset, which was downloaded by SEER∗Stat software (version 8.3.9.2), contained detailed clinical, demographic, and cause of death information for these patients. Clinical data consisted of abundant cancer-related diagnoses, including the primary site, histology subtype, tumour size and number, SEER stage, American Joint Committee on Cancer (AJCC) 6th edition tumour stage, and therapy (surgery, chemotherapy, and radiotherapy). The histologic definition of extremity osteosarcoma was based on World Health Organization's (WHO) categorisation [13]. Cases were selected as per the anatomic classification and documentation of the cancer in SEER based on the International Classification of Diseases for Oncology, Version 3 (ICD-03) [14].

Extremity osteosarcomas were defined by topography codes C40.0–40.3. Cases with histology codes 9180–9187 and 9193–9194 were included. All other topographies and morphologies were excluded from this research. Patients without data regarding survival endpoint were excluded. Patients whose race and ethnicity, SEER stage, tumour size, AJCC stage, tumour grade, and surgery were not clearly recorded were also excluded from the analysis.

**Figure 1:**
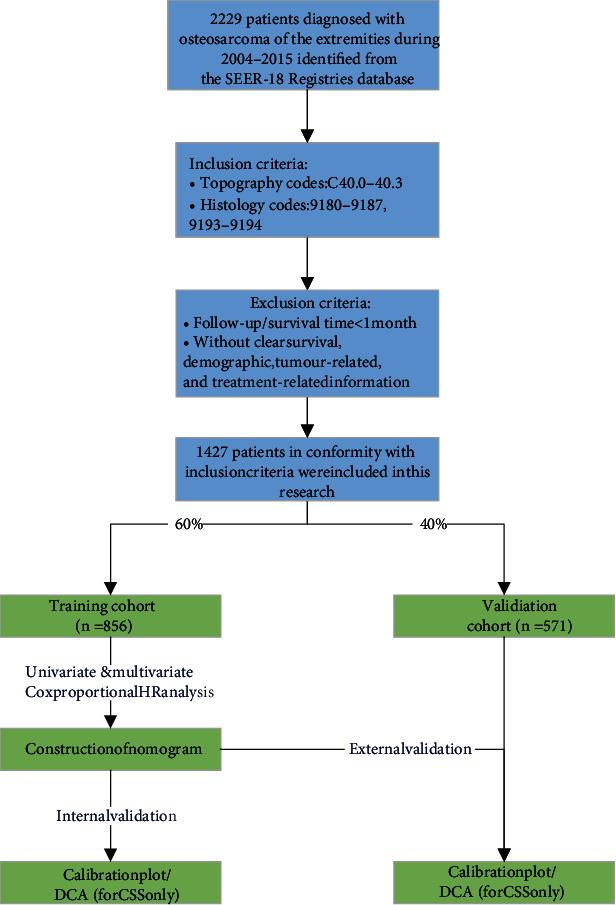
Flowchart of sample selection.

Eventually, a total of 1427 patients met the inclusion and exclusion criteria and were included in this study. The samples were randomly split into either the training set (*n* = 856) or the test cohort (*n* = 571) at a ratio of 6 : 4. Descriptive statistics were calculated for all baseline variables. The flowchart of sample selection is presented in Figure 1.

### 2.3. Independent Variables Assessed

Age at diagnosis, tumour size, and tumour number in SEER were reported as continuous variables. However, using these predictors as categorical variables was believed to be potentially more useful in clinical practice [15, 16]. Also, age (*P* < 0.001) and tumour size (*P* < 0.001) were found to individually have a nonlinear effect on survival in accordance with the analysis-of-variance (ANOVA) tables for the corresponding univariate Cox hazard ratio (HR) regression models. Consequently, the X-tile program developed by researchers from Stanford University [15] was used to discover appropriate cut-points in age and tumour size in this investigation.

The patients' demographic variables taken into account included age, sex, and race.

#### 2.3.1. Age

Incidence of osteosarcoma is found to follow a bimodal distribution with respect to age with peaks in children/adolescents and the elderly [17]. Hence, age was included in this study. X-tile-based optimal cut-point selection analysis of patient age reveals three distinct populations, namely, <22 years old, 22–52 years old, and >52 years old.

#### 2.3.2. Gender

Differences in malignant osteosarcomas survival by gender were also observed [18]. Therefore, gender could be a potential prognostic factor.

#### 2.3.3. Race and Ethnicity

Race-ethnicity variable was divided into the three following subcategories: White, Black, and other (American Indian/Alaska Native, Asian, or Pacific Islander).

The tumour information includes histology, primary location, tumour number, tumour size, SEER stage, AJCC stage, and tumour grade.

#### 2.3.4. Histology

The histology categories included in this research are 9180 (osteosarcoma, NOS (not otherwise specified)), 9181 (chondroblastic osteosarcoma), 9182 (fibroblastic osteosarcoma), 9183 (telangiectatic osteosarcoma), 9184 (osteosarcoma in Paget disease), 9185 (small cell osteosarcoma), 9186 (central osteosarcoma), 9187 (intraosseous well-differentiated osteosarcoma), 9192 (parosteal osteosarcoma), 9193 (periosteal osteosarcoma), and 9194 (high-grade surface osteosarcoma). Patients with codes 9194, 9184, 9185, and 9187 were grouped in “other” due to their small sample size.

#### 2.3.5. Primary Location

Primary site of the cancer was identified under the guidance of ICD-O-3 anatomic codes, and patients were stratified into two groups according to site of primary tumour location. C40.0 (long bones: upper limb, scapula, and associated joints) and C 40.1 (short bones of upper limb and associated joints) were combined into “upper limb”, and C40.2 (long bones of lower limb and associated joints) and C40.3 (short bones of lower limb and associated joints) into “lower limb”.

#### 2.3.6. Tumour Number

Tumour number was separated into two categories (single and multiple) according to the cut-point of 1.

#### 2.3.7. Tumour Size

Tumour size was determined using the length of the largest dimension. For this variable, X-tile identifies the optimal division of the cohort into two subgroups (≤13.9 cm and >13.9 cm).

#### 2.3.8. SEER Stage

The SEER database employs the “historic” staging system for cancers, which generally defines the extension of cancer at the time of diagnosis with classifications that are quite consistent over time [19]. For this variable, localized (entirely limited to the cortex of bone, or no break in periosteum), regional (extension beyond the primary organ to adjacent bone, cartilage, or lymph nodes), and distant (extension from the primary site to distant bone, or lung, or lymph nodes either by direct extension or by discontinuous metastasis) stages [20, 21] were included.

#### 2.3.9. AJCC Stage

The AJCC stage available for osteosarcoma of the extremities diagnosed after 2004 is based on the AJCC 6th edition staging system and was defined as either stage I, II, III, or IV as assigned by the TNM grouping for osteosarcoma.

It is notable that AJCC stage is derived from related information which is already entailed within tumour size and SEER histologic stage [22]. Therefore, it would be not included as a variable in the final model in order to reduce the potential risk of multicollinearity.

#### 2.3.10. Tumour Grade

Tumour grade reported in the SEER database was determined by the reporting pathologist for each lesion and was classified into one of the following categories: grade I, well differentiated; grade II, moderately differentiated; grade III, poorly differentiated; or grade IV, undifferentiated. Generally, grades I and II were recognised as low grade, and grades III and IV were regarded as high grade [22, 23].

The treatment information from the SEER database consists of surgery, radiation therapy, and chemotherapy.

#### 2.3.11. Surgery

Surgery was used as an unordered categorical variable in this study. The reference subgroup (no surgery) consisted of those who received no surgical therapy. The other subpopulations were defined according to the types of surgery that patients underwent (limb-preserving surgery and amputation).

#### 2.3.12. Radiation Therapy

Radiation therapy variable was classified into two mutually exclusive categories: radiation (yes) and no radiation/unknown.

#### 2.3.13. Chemotherapy

Chemotherapy variable was divided into two subgroups: chemotherapy (yes) and no chemotherapy/unknown.

### 2.4. Outcome Variables

#### 2.4.1. Survival Status and Time

Outcomes were OS time and CSS time and cause of death in this research. Survival time in SEER was measured in month. OS time was defined as the time from diagnosis to the date of death from any cause or the date of last known follow-up if death had not occurred. CSS time was defined as the time from the date of diagnosis to the date of death from the cancer or the date of last known follow-up if death had not occurred.

### 2.5. Statical Methods

All statical calculations in this investigation were performed using RStudio software for Windows (version: 1.4.1717; RStudio, Boston, MA), an integrated development environment (IDE) for the R programming language [24]. All tests were applied two-sided at a significance level of 5%.

In order to achieve the purpose of assessing the difference in patient characteristics between training and test cohorts, the Mann–Whitney *U* test was used for continuous variables, and the Pearson's chi-square test for categorical variables. Then, OS rates and CSS rates of the patients in the training set were measured at 1-, 3-, and 5-year postdiagnosis based on Kaplan–Meier (K–M) analyses using the *survival* package [25].

With the aid of the *survival* package [25], predictors of OS and CSS in the training cohort were evaluated by univariate and multivariate Cox proportional hazard models and summarized as HR. The HR values describe the relative ratio of the probability of a death event occurring in the comparison subgroup compared to the reference subcategory of each categorical variable of interest. As such, HR > 1.0 (<1.0) means a higher (lower) risk of a death event for patients in that subgroup versus the reference subcategory. 95% confidence interval (CI) for HR was also adopted.

Afterwards, variables having significant associations with OS and CSS of the training cohort in multivariable Cox analysis were employed to construct the nomograms for prediction of 1-, 3-, and 5-year OS and CSS probability of individual patients, respectively. The nomograms were separately subjected to both internal and external validation using the training and validation cohort. Besides, the *DynNom* package [26] was utilised to build web-based nomograms as a Shiny application.

The concordance between observed and predicted outcomes for the OS and CSS nomograms was evaluated using the concordance index (C-index) which ranges from 0.5 to 1. To assess the calibration and discrimination of the model corrected for optimism, 1000 bootstrap resamples were conducted using the *rms* package of R [27]. Specifically, a model was built on each of the 1000 simulated datasets that were generated by randomly selecting individuals from the original database in the bootstrap validation procedure. The 1000 models were subsequently evaluated on the original data without modification, estimating observed 1-, 3-, and 5-year OS/CSS probabilities for each patient. The calibration plots graphically display the correspondence between nomogram-predicted OS/CSS probability and observed actual rate of OS/CSS. A novel discriminative performance measurement method, decision curve analysis (DCA) was simultaneously employed to internally and externally validate the CSS nomogram. Decision curves were constructed using the *ggDCA* package [28]. With the help of DCA, the net benefit derived from the employment of the CSS nomogram model within the SEER population was compared to the net benefits obtained from the “intervention for all patients” and “intervention for none” options [29].

## 3. Results

### 3.1. Descriptive Statistics

Of the 1427 patients selected from the SEER database, 856 samples were used for prognostic nomogram development and 571 for external validation. Patient demographics and clinical characteristics of the entire dataset, training cohort, and test set were summarized in Table 1. The median age of patients diagnosed with osteosarcoma of the extremities in the study was 17 years [interquartile range (IQR): 13, 31]. 44.3% of the samples were male, and 55.7% were female. 74.5% were white, 16.1% black, and 9.4% other. Over 90% of patients were recorded as having undergone tumour resection, and 86.7% of samples as having undergone chemotherapy. 33.7% of lesions were localized to their primary site, and the left lesions demonstrate regional (45.4%) or distant (20.9%) invasion. Tumour grade was low grade (grade I or II) for 155 (10.9%) patients and high grade (grade III or IV) for the remaining 1,272 (89.1%) patients. As to AJCC staging, 1131 patients (79.3%) were stage I or II, and 296 patients (20.7%) were stage III or IV. There were no significant differences in the baseline characteristics between the two cohorts.

### 3.2. Survival Analyses

**Figure 2:**
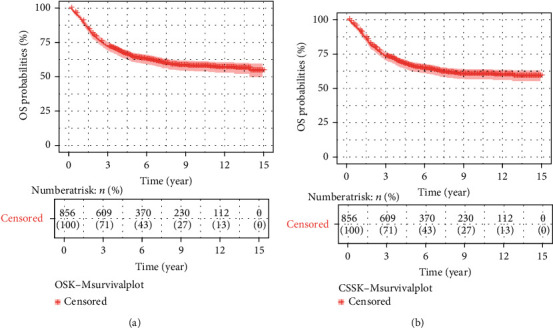
K–M survival plots for patients with extremity osteosarcoma showing (a) overall survival (OS) and (b) cancer-specific survival (CSS).

In the training cohort of 856 samples, 520 (60.7%) patients were reported alive at their last follow-up. Overall, 1-, 3-, and 5-year survival rates were 90.6% [95% CI: 88.7%–92.6%], 72.0% [95% CI: 69.0%–75.0%], and 64.0% [95% CI: 60.8%–67.4%], respectively (Table 2) (Figure 2(a)).

Among the 336 patients who died, 310 (92.3%) had death causes attributable to their osteosarcoma diagnosis. Cancer-specific 1-, 3-, 5-year survival rates were 91.4% [95% CI: 89.5%–93.3%], 73.3% [95% CI: 70.4%–76.4%], and 65.8% [95% CI: 62.6%–69.2%], respectively (Table 2) (Figure 2(b)).

### 3.3. Development and Validation of Nomogram for OS/CSS of Patients with Extremity Osteosarcoma

**Figure 3:**
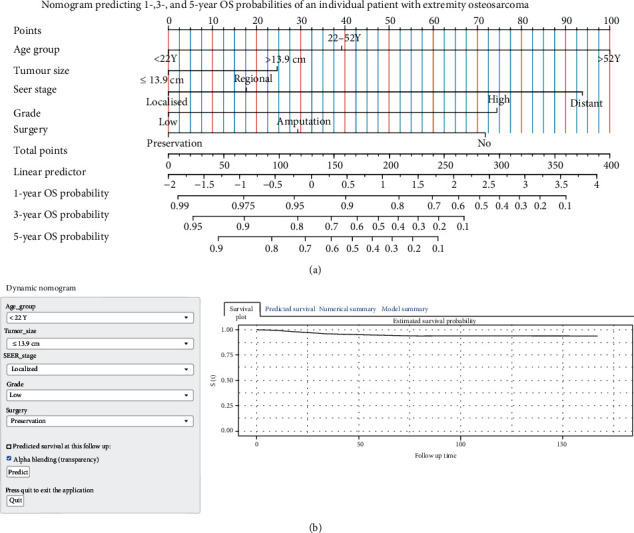
Nomogram predicting overall survival (OS) probabilities of patients with extremity osteosarcoma, and calibration plots for internal validation of the nomogram for the cumulative incidence of OS. (a) Nomogram predicting 1-, 3-, and 5-year OS probabilities of an individual patient with extremity osteosarcoma. The patient's value for each signature is plotted on its axis, and vertical lines are drawn to the axis of “points” (the first axis) to obtain the corresponding scores. All scores should be subsequently added to obtain the total point score. The total point score on the “total point” axis is printed in the seventh line, and a vertical line is drawn down to the three probability-predicting axes. The corresponding values present the predicted probability of 1-, 3-, and 5-year OS. The linear predictor axis represents how many linear predictor units there are per point and the number of points per unit change. (b) Web-based dynamic nomogram predicting 1-, 3-, and 5-year OS probabilities of an individual patient with extremity osteosarcoma (available at: https://tumournomo.shinyapps.io/Osteosarcoma_OS/).

**Figure 4:**
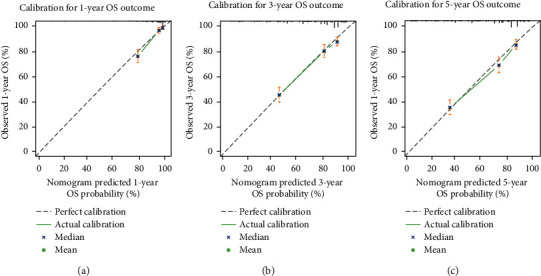
(a)–(c) Calibration plots of the OS nomogram within the training cohort predicting OS at 1 year, 3 years, and 5 years.

Table 3 shows the results of uni- and multivariable Cox HR analyses predicting OS. In univariable analyses, age categories, sex, AJCC stage, SEER stage, tumour size, histology, surgery, and radiation therapy were presented as predictors of OS (all *P* < 0.05). The HR of receiving radiation therapy compared to no radiation therapy was lower than 1. The underlying reason might be due to its small population of patients who had received radiation therapy (4.0%). These variables except for AJCC stage were included in the multivariable Cox HR regression model. Sex and radiation therapy lacked statistical significance in the multivariable analysis, and the other variables were included in the nomogram.  Figure 3(a) displays the nomogram that can be utilised to predict 1-, 3-, and 5-year OS probability of an individual patient with extremity osteosarcoma using independent prognosis signatures. The C-index of the nomogram was 0.752 [95% CI: 0.738–0.765].  Figure 3(b) shows the image of the web-based nomogram available at https://tumournomo.shinyapps.io/Osteosarcoma_OS/. A dynamic graphical calculating of that kind is very simple-to-use and permits a clinician to interact with the statistical model in a user-friendly manner [30]. The nomogram displays a pull-down box for each categorical covariate. In a calibration plot, the *X*-axis stands for the nomogram-predicted OS probability, and its *Y*-axis represents the observed outcome. Predictions indicating ideal results should be on the 45° line [31]. Figures 4(a)–4(c) demonstrate that this model had good calibration.

**Figure 5:**
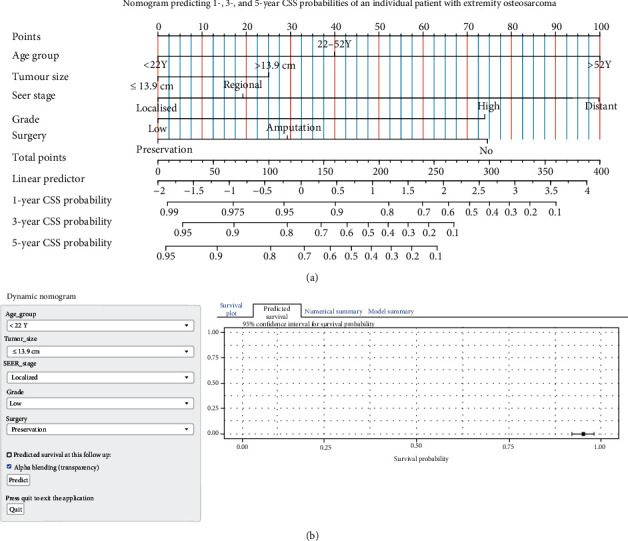
Nomogram predicting cancer-specific survival (CSS) probabilities of patients with extremity osteosarcoma, and calibration plots for internal validation of the nomogram for the cumulative incidence of CSS. (a) Nomogram predicting 1-, 3-, and 5-year CSS probabilities of an individual patient with extremity osteosarcoma. The patient's value for each signature is plotted on its axis, and vertical lines are drawn to the axis of “points” (the first axis) to obtain the corresponding scores. All scores should be subsequently added to obtain the total point score. The total point score on the “total point” axis is printed in the seventh line, and a vertical line is drawn down to the three probability-predicting axes. The corresponding values present the predicted probability of 1-, 3-, and 5-year CSS. The linear predictor axis represents how many linear predictor units there are per point and the number of points per unit change. (b) Web-based dynamic nomogram predicting 1-, 3-, and 5-year OS probabilities of an individual patient with extremity osteosarcoma (available at: https://tumournomo.shinyapps.io/Osteosarcoma_CSS/).

**Figure 6:**
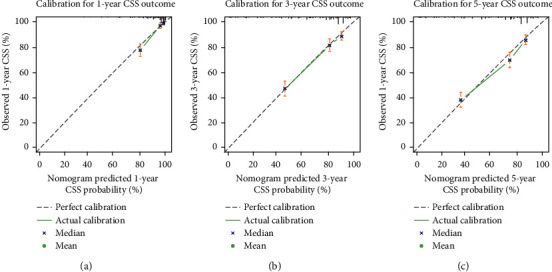
(a)–(c) Calibration plots of the CSS nomogram within the training cohort predicting CSS at 1 year, 3 years, and 5 years.

As indicated in Table 4, univariable Cox HR regression for CSS probabilities found radiation therapy and surgery (all *P* < 0.05) to be statistically significant in predicting survival. Older age (*P* < 0.05) was significantly associated with an increased hazard of death. The other prognosticators showed similar trends: male gender, primary tumour size > 13.9 cm, AJCC stages III–IV, high tumour grade, and tumour distant extension (all *P* < 0.05). These variables other than AJCC stage were included in the multivariable Cox HR regression model. Similar to OS, sex and radiation therapy did not have a statistically significant impact on CSS in the multivariable analysis. As depicted in Figure 5(a), the estimated parameters of variables having significant effects on CSS were used to construct the nomogram-based CSS prediction tool.  Figure 5(b) shows the dynamic version of the nomogram available at https://tumournomo.shinyapps.io/Osteosarcoma_CSS/. The assessment process of nomogram-predicted 1-, 3-, and 5-year CSS probability of an individual patient with extremity osteosarcoma using (Figure 5(a) and 5(b)) is similar to the method applied to Figures 3(a) and 3(b). The web-based predictive tools can aid personalised treatment and clinical decision-making. The C-index of the nomogram was 0.754 [95% CI: 0.740–0.768]. Figures 6(a)–6(c) imply that there were only minor deviations from perfect agreement between predicted and actual CSS probabilities.

**Figure 7:**
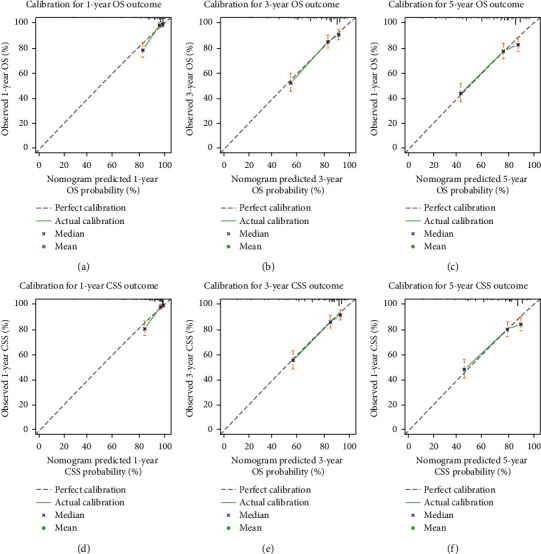
Calibration plots for external validation of the nomogram within the test cohort for cumulative incidence of overall survival (OS) and cancer-specific survival (CSS). Observed cumulative incidence is plotted against nomogram-predicted cumulative incidence. (a)–(c) Calibration plots of the OS nomogram within the test cohort predicting OS at 1 year, 3 years, and 5 years. (d)–(f) Calibration plots of the CSS nomogram within the test cohort predicting CSS at 1 year, 3 years, and 5 years.

External validation showed that the nomogram accurately predicted OS/CSS when applied to an independent test cohort of 571 patients. The OS/CSS nomogram was validated on the test cohort with C-indexes of 0.720 [95% CI: 0.701–0.738] and 0.727 [95% CI: 0.707–0.746], and both of them were well calibrated with respect to the observed outcome as shown in Figure 7.

**Figure 8:**
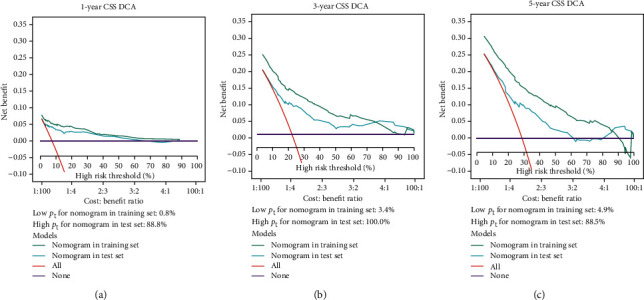
Decision curves plotting net benefit against threshold probability. (a)–(c) Decision curve analyses (DCA) of nomogram model within the training cohort (green line) and the test set (blue line) predicting cancer-specific survival (CSS) at 1 year, 3 years, and 5 years. *p*_*t*_ refers to the point where the expected benefit of intervention is equal to the expected benefit of avoiding intervention.

Intervention reflecting any action that a patient getting a favourable result on a diagnostic test would be taken into consideration to improve their life in general [32]. In a DCA plot, clinical usefulness is displayed where a model has greater net benefit than an “all (intervention for all patients)” or “none (intervention for none)” approach [33]. Decision curves in (Figures 8(a)–8(c)) show that the 1-, 3-, and 5-year net benefits of the CSS nomogram model established on the training cohort (green line) were greater than the “all” and “none” options at threshold probabilities greater than 4.9% and less than 88.5%. These figures also demonstrate that the CSS nomogram model established on the test set (blue line) also performed well in DCA. It can be concluded that using the model to predict survival rates of patients with extremity osteosarcoma would lead to improved clinical outcome.

## 4. Discussion

The employment of validated nomograms has become more popular in clinical practice due to their precision and usability. Therefore, there is indeed a need to identify their predictive accuracy and easiness of use in the development of predictive models for survival in patients with cancer. In this historical cohort study of osteosarcoma patients, five significant independent prognosis factors were identified and were used to construct validated nomograms for predicting 1-, 3-, and 5-year OS/CSS rates. Only patients with extremity osteosarcoma were analysed here, since extremity osteosarcoma is the most common primary osteosarcoma, and relatively few predictive models were developed for clinical outcomes of patients with the cancer at present.

### 4.1. Independent Prognostic Signatures

In this SEER database analysis, advanced age was found to be significantly associated with worse prognosis. The research of Longhi [34] also revealed that patients older than 65 years with osteosarcoma usually have a worse survival outcome compared to younger age group. The authors further explained that the older age group features a longer period from the commencement of symptoms to diagnosis, more metastatic cases at diagnosis, and less employment of extremity salvage, and more patients were excluded from clinical treatment trials than younger patients [34].

Besides, the greatest dimension of an extremity osteosarcoma is considered an important prognostic factor, which concurs with previous reports. The research of Bielack et al. [35] revealed that osteosarcoma progression is significantly associated with larger tumour size. Tumours whose volume is larger than 371 cm^3^ may have a two times greater incidence of lung metastasis [36]. There was also evidence indicating that an increase in tumour size during neoadjuvant chemotherapy is predictive of local recurrence [37].

In the meantime, patients with lower grade appear to have improved survival. It has been noted by Holden et al. [38] that patients with well-differentiated tumours seem to survive longer. A possible explanation for this may be that low-grade osteosarcomas are related to a lower incidence of metastases [39]. In addition to that, some studies indicated that high-grade osteosarcoma of the extremity treated only with conventional surgery could lead to a case fatality rate of 90% [40, 41].

Our research also implies the risk of death is higher for those patients whose tumours have spread to distant sites. 15%–20% of osteosarcoma patients have detectable metastasis at diagnosis, 85–90% of which occurs in the lungs [5, 42]. Furthermore, lung metastasis from osteosarcomas is a primary cause of death [36].

Moreover, the presence of surgery can make an impact on patients' survival. Most patients with osteosarcoma having undergone extremity salvage surgery instead of amputation during the period of chemotherapy can keep the majority of their limb functions, as well as improve their quality of life [43]. This also suggests that surgeons should continue to strive to salvage as much of the limb as possible when performing tumour resections [44].

On the other hand, our findings indicate radiation therapy is not an independent prognostic factor for patients' OS and CSS. The study of Hız et al. [45] showed that preoperative radiotherapy could offer increased rates of necrosis and excision of unresectable lesions in the treatment of osteosarcoma but did not significantly enhance patients' 5-year survival.

### 4.2. Limitations

Although the SEER database offers clinicians the opportunity to conduct a retrospective study with a considerable amount of population, there are still some limitations to be acknowledged. Over the years, staging schemes have changed. In 2017, AJCC released its 8th edition *Cancer Staging Manual* [46]. However, it could be hard to reevaluate the data with the latest standard. Besides, the sample size of patients who had received radiation therapy was relatively small in this cohort. As to surgical treatment, the extent of resection and margin status of resection specimens is not available. Apart from the abovementioned restrictions, future studies should externally validate the SEER population-based predictive models in patients from different clinics.

## 5. Conclusions

In conclusion, the population data from the SEER-Medicare database can be useful for assessing prognosis in patients with osteosarcoma of the extremities. In the training cohort of 856 samples, 39.3% of patients died at or before their last follow-up, over 90% of whom died from cancer-specific causes. 5-year OS and CSS rates were found to be 64.0% and 65.8%, respectively, which were at the higher end of what was previously reported in research articles discussing the survival prediction of osteosarcoma [47–49].

Based on exploratory data analysis, patients with increased tumour size, higher grade, distant tumour extension, amputation, or no surgery were considered to be at significantly greater risk of overall and cancer-specific death at any time point of the disease course. Prior to 1970, amputation was the only available surgical treatment for osteosarcoma patients [50, 51]. The result implies that the reduction in mortality may be attributed to the development of surgical techniques. The nomogram-based survival prediction tools, which demonstrated good accuracy in both internal and external validation, can serve as a counselling and clinical decision aid to patients for clinicians.

## Figures and Tables

**Table 1:** Demographics, tumour characteristics, and treatment characteristics of patients with extremity osteosarcoma.

Characteristics	Overall (*n* = 1427)	Training cohort (*n* = 856)	Test cohort (*n* = 571)	*P*
Age at diagnosis (median [IQR])	17 [13, 31]	17 [12, 31]	17 [13, 32]	0.578^a^
Sex (%)				
Female	632 (44.3)	366 (42.8)	266 (46.6)	0.170^b^
Male	795 (55.7)	490 (57.2)	305 (53.4)	
Race and ethnicity (%)				
White	1063 (74.5)	634 (74.1)	429 (75.1)	0.576^b^
Black	230 (16.1)	136 (15.9)	94 (16.5)	
Other	134 (9.4)	86 (10.0)	48 (8.4)	
Primary site (%)				
Upper limb	233 (16.3)	133 (15.5)	100 (17.5)	0.360^b^
Lower limb	1194 (83.7)	723 (84.5)	471 (82.5)	
Histology (%)				
9180	899 (63.0)	527 (61.6)	372 (65.1)	0.537^b^
9181	190 (13.3)	119 (13.9)	71 (12.4)	
9182	76 (5.3)	50 (5.8)	26 (4.6)	
9183	61 (4.3)	41 (4.8)	20 (3.5)	
9186	55 (3.9)	29 (3.4)	26 (4.6)	
9192	90 (6.3)	56 (6.5)	34 (6.0)	
Other	56 (3.9)	34 (4.0)	22 (3.9)	
Tumour size (median [IQR])	9.300 [6.850, 13.000]	9.200 [7.000, 13.000]	9.500 [6.500, 13.000]	0.733^a^
Tumour number (median [IQR])	1.000 [1.000, 1.000]	1.000 [1.000, 1.000]	1.000 [1.000, 1.000]	0.600^a^
AJCC stage (%)				
Stage I	145 (10.2)	95 (11.1)	50 (8.8)	0.270^b^
Stage II	986 (69.1)	580 (67.8)	406 (71.1)	
Stage III	24 (1.7)	12 (1.4)	12 (2.1)	
Stage IV	272 (19.1)	169 (19.7)	103 (18.0)	
Seer stage (%)				
Localized	481 (33.7)	299 (34.9)	182 (31.9)	0.430^b^
Regional	648 (45.4)	378 (44.2)	270 (47.3)	
Distant	298 (20.9)	179 (20.9)	119 (20.8)	
Tumour grade (%)				
Grade I	58 (4.1)	39 (4.6)	19 (3.3)	0.282^b^
Grade II	97 (6.8)	64 (7.5)	33 (5.8)	
Grade III	422 (29.6)	257 (30.0)	165 (28.9)	
Grade IV	850 (59.6)	496 (57.9)	354 (62.0)	
Surgery (%)				
Preservation	1010 (70.8)	610 (71.3)	400 (70.1)	0.513^b^
Amputation	326 (22.8)	188 (22.0)	138 (24.2)	
No	91 (6.4)	58 (6.8)	33 (5.8)	
Chemotherapy (%)				
Yes	1237 (86.7)	739 (86.3)	498 (87.2)	0.688^b^
No/unknown	190 (13.3)	117 (13.7)	73 (12.8)	
Radiation therapy (%)				
Yes	57 (4.0)	36 (4.2)	21 (3.7)	0.718^b^
No/unknown	1370 (96.0)	820 (95.8)	550 (96.3)	

IQR: Interquartile range. ^a^*P* value was obtained from the Mann–Whitney *U* test. ^b^*P* value was obtained from the Pearson's chi-square test.

**Table 2:** 1-, 3-, and 5-year overall survival (OS) and cancer-specific survival (CSS) probabilities of patients with extremity osteosarcoma as estimated by K–M analyses.

Time	OS probability [95% CI]	CSS probability [95% CI]
1-year	90.6% [88.7%–92.6%]	91.4% [89.5%–93.3%]
3-year	72.0% [69.0%–75.0%]	73.3% [70.4%–76.4%]
5-year	64.0% [60.8%–67.4%]	65.8% [62.6%–69.2%]

CI: Confidence Interval.

**Table 3:** Uni- and multivariable Cox hazard ratio (HR) analyses for overall survival (OS) in patients with extremity osteosarcoma.

Characteristics	Univariable Cox HR regression			Multivariable Cox HR regression		
	HR	95% CI	*P*	HR	95% CI	*P*
Age at diagnosis						
<22	1	Reference		1	Reference	
22–52 Y	1.397	[1.089–1.792]	0.008	1.903	[1.469–2.465]	*P* < 0.001
> 52 Y	4.255	[3.184–5.688]	*P* < 0.001	4.67	[3.412–6.391]	*P* < 0.001
Sex						
Female	1	Reference		1	Reference	
Male	1.516	[1.211–1.898]	*P* < 0.001	1.21	[0.96–1.526]	0.107
Race and ethnicity						
White	1	Reference				
Black	1.074	[0.804–1.436]	0.629			
Other	1.092	[0.766–1.556]	0.627			
Primary site						
Upper limb	1	Reference				
Lower limb	1.079	[0.795–1.465]	0.625			
Histology						
9180	1	Reference		1	Reference	
9181	0.861	[0.631–1.175]	0.346	1.052	[0.766–1.445]	0.754
9182	0.803	[0.508–1.268]	0.346	0.768	[0.479–1.233]	0.274
9183	0.676	[0.394–1.159]	0.155	0.627	[0.363–1.085]	0.095
9186	0.379	[0.169–0.853]	0.019	0.488	[0.216–1.103]	0.085
9192	0.199	[0.088–0.447]	*P* < 0.001	0.515	[0.197–1.347]	0.176
Other	0.631	[0.335–1.188]	0.154	0.675	[0.356–1.279]	0.228
Tumour size						
≤13.9 cm	1	Reference		1	Reference	
> 13.9 cm	1.788	[1.407–2.271]	*P* < 0.001	1.494	[1.167–1.912]	0.001
Tumour number						
Single	1	Reference				
Multiple	0.797	[0.566–1.121]	0.193			
AJCC stage						
Stages I–II	1	Reference				
Stages III–IV	4.281	[3.43–5.343]	*P* < 0.001			
SEER stage						
Localized	1	Reference		1	Reference	
Regional	1.525	[1.146–2.029]	0.004	1.313	[0.983–1.753]	0.066
Distant	5.38	[4.031–7.182]	*P* < 0.001	4.024	[2.963–5.465]	*P* < 0.001
Grade						
Low grade	1	Reference		1	Reference	
High grade	3.477	[2.071–5.835]	*P* < 0.001	2.363	[1.274–4.383]	0.006
Surgery						
Preservation	1	Reference		1	Reference	
Amputation	1.853	[1.456–2.357]	*P* < 0.001	1.594	[1.244–2.044]	*P* < 0.001
No	4.442	[3.157–6.25]	*P* < 0.001	2.843	[1.935–4.177]	*P* < 0.001
Chemotherapy						
Yes	1	Reference				
No/unknown	0.724	[0.508–1.032]	0.074			
Radiation						
Yes	1	Reference		1	Reference	
No/unknown	0.321	[0.215–0.48]	*P* < 0.001	0.789	[0.496–1.255]	0.317

CI: Confidence interval.

**Table 4:** Uni- and multivariable Cox hazard ratio (HR) analyses for cancer-specific survival (CSS) in patients with extremity osteosarcoma.

Characteristics	Univariable Cox HR regression			Multivariable Cox HR regression		
	HR	95% CI	*P*	HR	95% CI	*P*
Age at diagnosis						
<22	1	Reference		1	Reference	
22–52 Y	1.393	[1.077–1.801]	0.012	1.903	[1.456–2.487]	*P* < 0.001
> 52 Y	3.847	[2.821–5.245]	*P* < 0.001	4.154	[2.972–5.806]	*P* < 0.001
Sex						
Female	1	Reference		1	Reference	
Male	1.449	[1.149–1.828]	0.002	1.152	[0.906–1.465]	0.247
Race and ethnicity						
White	1	Reference				
Black	1.076	[0.796–1.454]	0.636			
Other	1.041	[0.715–1.515]	0.836			
Primary site						
Upper limb	1	Reference				
Lower limb	1.149	[0.83–1.591]	0.402			
Histology						
9180	1	Reference		1	Reference	
9181	0.812	[0.583–1.13]	0.217	0.977	[0.698–1.369]	0.894
9182	0.781	[0.483–1.264]	0.314	0.771	[0.469–1.268]	0.306
9183	0.732	[0.426–1.257]	0.259	0.689	[0.398–1.193]	0.184
9186	0.409	[0.182–0.921]	0.031	0.521	[0.23–1.178]	0.117
9192	0.179	[0.074–0.435]	*P* < 0.001	0.434	[0.154–1.222]	0.114
Other	0.682	[0.362–1.285]	0.236	0.737	[0.388–1.4]	0.351
Tumour size						
≤13.9 cm	1	Reference		1	Reference	
> 13.9 cm	1.816	[1.417–2.327]	*P* < 0.001	1.495	[1.157–1.931]	0.002
Tumour number						
Single	1	Reference				
Multiple	1.126	[0.748–1.695]	0.568			
AJCC stage						
Stage I–II	1	Reference				
Stage III–IV	4.557	[3.624–5.728]	*P* < 0.001			
SEER stage						
Localized	1	Reference		1	Reference	
Regional	1.547	[1.144–2.092]	0.005	1.345	[0.991–1.827]	0.057
Distant	5.767	[4.265–7.798]	*P* < 0.001	4.327	[3.145–5.955]	*P* < 0.001
Grade						
Low grade	1	Reference		1	Reference	
High grade	3.425	[2.003–5.854]	*P* < 0.001	2.197	[1.169–4.127]	0.014
Surgery						
Preservation	1	Reference		1	Reference	
Amputation	1.826	[1.42–2.349]	*P* < 0.001	1.569	[1.211–2.034]	0.001
No	4.565	[3.213–6.486]	*P* < 0.001	2.89	[1.946–4.294]	*P* < 0.001
Chemotherapy						
Yes	1	Reference				
No/unknown	0.715	[0.493–1.036]	0.076			
Radiation						
Yes	1	Reference		1	Reference	
No/unknown	0.322	[0.212–0.488]	*P* < 0.001	0.799	[0.493–1.296]	0.363

CI: Confidence interval.

## Data Availability

The data used and analysed in this study are available in the Surveillance, Epidemiology, and End Results (SEER) Database of the National Cancer Institute (http://seer.cancer.gov).
